# Variante en gen *HARS* detectada en exoma clínico: etiología de neuropatía periférica tras más de 20 años sin diagnóstico

**DOI:** 10.1515/almed-2020-0020

**Published:** 2020-05-19

**Authors:** Raquel Lahoz Alonso, Paula Sienes Bailo, Jose Luis Capablo Liesa, Sara Álvarez de Andrés, Jose Luis Bancalero Flores, Silvia Izquierdo Álvarez

**Affiliations:** Servicio de Bioquímica Clínica, Hospital Universitario Miguel Servet, Zaragoza, España; Servicio de Neurología, Hospital Universitario Miguel Servet, Zaragoza, España; NIMGenetics, Madrid, España

**Keywords:** Charcot-Marie-Tooth, exoma, gen *HARS*

## Abstract

**Objetivos:**

Describimos un caso con enfermedad de Charcot Marie Tooth axonal tipo 2W, trastorno neurólogico caracterizado por una neuropatía periférica, que afecta principalmente a las extremidades inferiores y provoca dificultades en la marcha y deterioro sensitivo-motor distal.

**Presentación del caso:**

Es un caso en el que la aplicación de las nuevas técnicas de secuenciación masiva (NGS) a través del exoma clínico en los laboratorios de genética permitió detectar la presencia de variantes candidatas de la clínica del paciente.

**Conclusiones:**

La variante detectada en el gen HARS podría apoyar la causalidad en el contexto clínico del paciente tras 20 años sin diagnóstico y con empeoramiento de la clínica.

## Introducción

La enfermedad de Charcot-Marie-Tooth (CMT) comprende un grupo heterogéneo de enfermedades hereditarias que afectan a los nervios periféricos. Con una prevalencia de 1/2500 individuos, generalmente se manifiesta en la adolescencia o al comienzo de la edad adulta con debilidad muscular progresiva y atrofia en las extremidades, pérdida sensorial, deformidades esqueléticas, afectación de la marcha y ausencia de reflejos, aunque la intensidad de los síntomas es muy variable incluso en pacientes con una misma mutación [1].

Neurofisiológicamente se subdivide en tres tipos: CMT1, predominantemente desmielinizante con velocidades de conducción (VC) lentas de 38 m/s o menos en extremidades superiores (EESS); CMT2, predominantemente axonal con VC conservadas, pero amplitudes de los potenciales de acción musculares reducidas como consecuencia de la degeneración axonal y CMT intermedia con características desmielinizantes y axonales y VC de 25 a 45 m/s. Actualmente no existe tratamiento curativo para CMT y el manejo de los pacientes se centra en la terapia física y ocupacional, así como en la inmovilización con férulas, dispositivos ortopédicos y cirugía ortopédica para abordar los síntomas incapacitantes de la enfermedad [2].

La forma de herencia puede ser autosómica dominante, autosómica recesiva o ligada al X [3]. Hasta la fecha, más de 80 genes se han relacionado con CMT [4]. Centrándonos en la CMT2, mutaciones en el gen de la mitofusina 2 (*MNF2*) se asocian con el subtipo CMT2A mayoritario (10–30% de las mutaciones) [5], pero se estima que en más de tres cuartas partes de los casos no se logra elucidar el origen genético de la enfermedad debido a la gran heterogeneidad génica, pleiotropía y ausencia de otros genes responsables de una proporción sustancial de los casos [3, 6, 7].

Entre los genes relacionados con CMT se han identificado a día de hoy cinco que codifican diferentes aminoacil-ARNt sintetasas (ARSs), enzimas encargadas de unir a moléculas de ARNt afines los aminoácidos específicos que serán transferidos a péptidos en crecimiento durante el proceso de traducción en el ribosoma: glicil-(GARS), tirosil-(YARS), alanil-(AARS), triptofanil-(WARS), histidil-ARNt sintetasa (HARS) [8], [9], [10], [11], [12]. En el caso del gen *HARS*, estudios previos evidenciaban la relación existente entre mutaciones de este gen y la enfermedad de CMT [13], cuya fisiopatología ha sido vinculada en otros más recientes a la reducción de la actividad catalítica de la enzima HARS a la que conducen dichas mutaciones [14, 15].

## Caso clínico

Varón de 34 años, español, derivado a la Consulta de Genética Clínica del Hospital Universitario Miguel Servet (HUMS) (Zaragoza, España) por cuadro de polineuropatía axonal motora con leve o dudosa alteración sensitiva.

A la edad de 8 años se objetivó una elevación de la inmunoglobulina E total (IgE, 560 UI/mL), que se mantuvo con los años alcanzado niveles máximos de 2484 UI/mL, descartándose problemas alérgicos y parásitos intestinales. A los 23 años presentaba polineuropatía periférica progresiva que afectaba a ambas extremidades inferiores (EEII).

Los estudios neurofisiológicos mostraron la presencia de severa denervación muscular y una neuropatía axonal motora en ambas EEII por afectación distal muy predo-minante y distribución bilateral sin asimetrías con estudios sensitivos normales en EESS y con muy leves anomalías en EEII. Desde los 14 años se objetivaban pies cavos y dedos en martillo con limitaciones para correr y caminar deprisa, y dolores en pies y piernas. No tenía antecedentes fami-liares de interés salvo su madre, que refiere temblores.

Ante la sospecha de una posible neuropatía periférica hereditaria de tipo CMT se solicitó un panel de Next Generation Sequencing (NGS) de 34 genes asociado a CMT (Sistemas Genómicos, ASCIRES, Valencia, España) (Material suplementario, Anexo complementario 1). No se detectó ninguna variante claramente patogénica asociada a CMT en ninguno de los genes incluidos en el panel, a excepción de la presencia de una variante de significado incierto (VSI) en el gen *KARS* (Tabla 1), de acuerdo con la clasificación propuesta en el documento American College of Medical Genetics and Genomics (ACMG) standards and guidelines for the interpretation of sequence variants (Genetics in Medicine, 2015). Esta variante no está descrita en las bases Human Gene Mutation Database (HGMD) y Leiden Open Variation Database (LOVD) y en la base de datos Database of single nucleotide polymorphism (dbSNP) ha sido identificada con una frecuencia asociada al alelo minoritario de 0,1%. Por otro lado, los estudios *in silico* no muestran una alteración clara del splicing debido a la presencia de esta variante que recientemente ha sido catalogada como benigna por varios autores en la base de datos ClinVar. No obstante, debido a la ausencia de correlación con el fenotipo del paciente y al carácter recesivo de la patología a la que se asocian las mutaciones en este gen, aun confirmándose la patogenicidad de dicha variante, sería necesaria la presencia de otra alteración para poder confirmar, desde un punto de vista genético, su cuadro clínico.

**Tabla 1:** Estudios genéticos realizados en el paciente y sus progenitores.

	Gen	Nomenclatura variante	Exón	Cigosidad	Efecto	Categorización variante	Herencia	Fenotipo
A	*KARS*	c.696A>G p.(Thr232Thr)	6	Het	UD	VUS	AR	CMT intermedia tipo B
B	*HARS*	c.397G>T p.(Val133Phe)	5	Het	*missense*	VUS	AD	CMT axonal tipo 2W
C	*MUTYH*	c.1187G>A p.(Gly396Asp)	13	Het	*missense*	VP	AR	Poliposis adenomatosa familiar
D	*HARS*	c.397G>T p.(Val133Phe)	5	Het	*missense*	VUS	AD	CMT axonal tipo 2W
E	*MUTYH*	c.1187G>A p.(Gly396Asp)	13	Hom	*missense*	VP	AR	Poliposis adenomatosa familiar

Het, heterocigosis; Hom, homocigosis; UD, indeterminado; VUS, variante de significado incierto; VP, variante patogénica; AR, autosómica recesiva; AD, autosómica dominante. Variantes genómicas identificadas en: (A) panel de 34 genes asociado a CMT en el paciente (Sistemas Genómicos, ASCIRES, Valencia, España) y (B) exoma clínico en sangre periférica (ExoNIM^®^ NIMGenetics). Hallazgo incidental encontrado en el exoma clínico del paciente según la recomendación de la American College of Medical Genetics and Genomics (ACMG) (C). Evaluación del patrón de segregación de las variantes descritas en el paciente mediante secuenciación Sanger: (D) en la madre (Cerba Internacional, Sabadell, Barcelona) y (E) en el padre (NIMGenetics, Madrid, España).

Se realizaron nuevos estudios de electroneurografía y electromiografía en los que se confirmó la sospecha de neuropatía axonal con intensa afectación de los troncos nerviosos motores de EEII sin afectar a EESS. La norm-alidad de las VC y latencias distales descartaron que se tratara de un caso de neuropatía desmielinizante y en la musculatura subsidiaria se identificó un patrón neurógeno con intensos signos de denervación y pérdida de unidades motoras. En nervios sensitivos, la amplitud se encontró en el límite inferior dentro del rango de normalidad.

**Figura 1:**
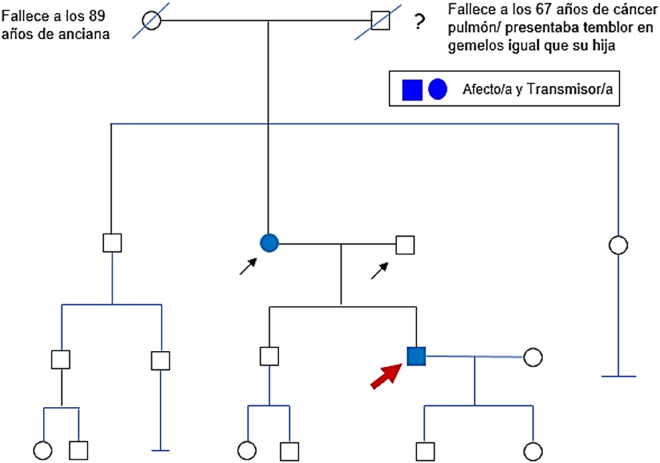
Pedigrí. Las flechas señalan las personas de la familia estudiadas.

Se amplió el estudio genético por NGS con la secuenciación del exoma clínico (ExoNIM^®^ NIMGenetics, Madrid, España). De esta forma se identificó la variante descrita en la Tabla 1 en heterocigosis, previamente descrita con una sola evidencia en la base de datos ClinVar (Variation ID: 548118) como una variante probablemente patogénica asociada con la enfermedad de CMT axonal tipo 2W (MIM#616625). Asimismo, en la base de datos HGMD, se describen variantes patogénicas de cambio de sentido asociadas a neuropatía periférica en codones adyacentes. Además, se encontró como hallazgo incidental una variante patogénica en el gen *MUTYH* asociada a poliposis adenomatosa familiar autosómica recesiva (Tabla 1). Se evaluó mediante secuenciación Sanger el patrón de segregación familiar de las variantes *HARS* c.397G>T; p.(Val133Phe) y *MUTYH* c.1187G>A; p.(Gly396Asp), en los progenitores para establecer si se trataban de variantes *de novo* o heredadas (Figura 1).

El padre fue identificado como portador de la variante *MUTYH* c.1187G>A; p.(Gly396Asp) en homocigosis, confirmando la herencia por línea paterna de dicha variante (Tabla 1) (NIMGenetics, Madrid, España). En la madre, con electromiograma alterado, se evidenció la presencia de la variante c.397G>T; p.(Val133Phe) en heterocigosis en el gen *HARS*, confirmando su herencia por línea materna (Tabla 1) (Cerba Internacional, Sabadell, Barcelona, España) aunque no fue posible ampliar el estudio a otros familiares.

**Figura 2:**
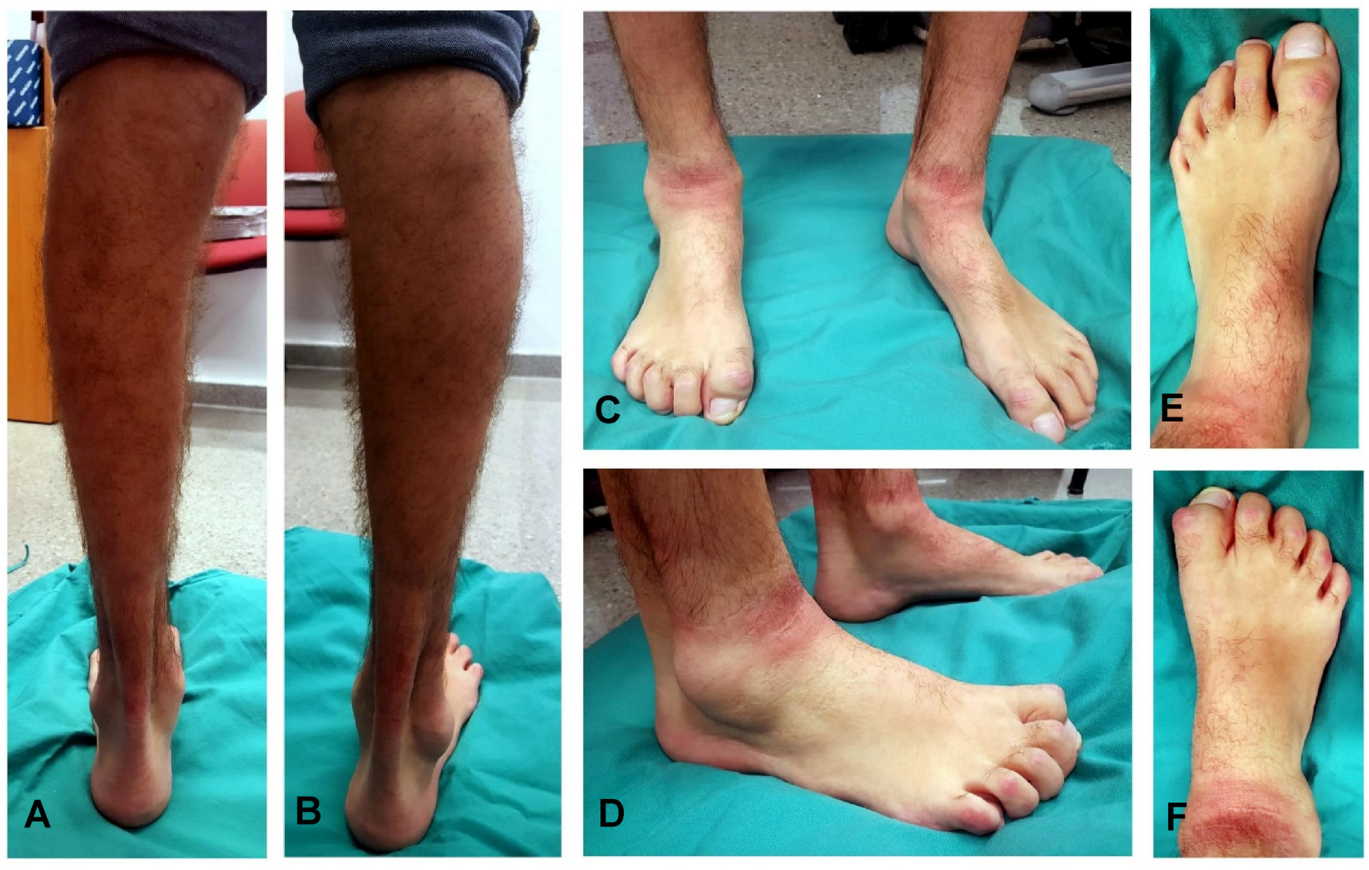
Fenotipo clínico del paciente. En (A) pierna izquierda y (B) pierna derecha se evidencia la atrofia gemelar. En (C–D) se muestran imágenes frontal y lateral de ambos pies. En el izquierdo (E) se observa un leve flexo dorsal. En el derecho (F) no existe flexión dorsal, el primer dedo se muestra rígido con un callo dorsal y el segundo es también casi rígido.

En la actualidad, el paciente presenta pie neurológico con tendencia a garra en los dedos primero al quinto, atrofia en los gemelos y refiere cansancio y pérdida de fuerza en EEII (Figura 2). Se encuentra en tratamiento con curcumina y dieta realizando periodos de ayuno. Este tratamiento ha demostrado incrementar el número y tamaño de axones mielinizados y mejorar el rendimiento motor en ratones Tembler-J [16]. Además, utiliza plantillas y una tobillera antiequino y posee un plan de ejercicios adaptado.

## Discusión

La presencia de variantes en *HARS* se asocia, con un patrón de herencia autosómico dominante, a la enfermedad de CMT axonal tipo 2W (MIM#616625). La enfermedad de CMT tipo 2W es un trastorno neurológico caracterizado por una neuropatía periférica, que afecta principalmente a las EEII y provoca dificultades en la marcha y deterioro sensorial distal, aunque la mayoría de los pacientes también tienen afectación de las EESS. En nuestro caso, la falta de afectación sensitiva y la ausencia de manifestaciones a nivel de EESS puede deberse a la juventud del paciente, dado que el trastorno sensorial distal puede aparecer con la edad o con una forma clínica de presentación como neuropatía motora pura que se considera un fenotipo diferenciado de esta enfermedad.

La variante Val133Phe identificada en heterocigosis en nuestro paciente es una variante de cambio de sentido que ha sido previamente descrita con una sola evidencia en la base de datos ClinVar como una variante probablemente patogénica. Esta mutación fue reportada por primera vez por Royer-Bertrand et al. en 2019 [14], quienes comprobaron el efecto deletéreo de esta variante sobre la función de la HARS al afectar a aminoácidos altamente conservados del dominio catalítico, provocando una reducción de la síntesis de histidil-ARNt sin verse alterada la expresión de esta proteína en las células.

Asimismo, en las bases de datos HGMD, ClinVar y UniprotKB se han descrito variantes patogénicas de cambio de sentido asociadas a neuropatía periférica en codones adyacentes ([c.395C>T p.(Thr132Ile), c.401C>A p.(Pro134His)]) [12, 13], lo que refuerza la implicación de esta variante en el cuadro clínico de nuestro paciente.

**Figura 3:**
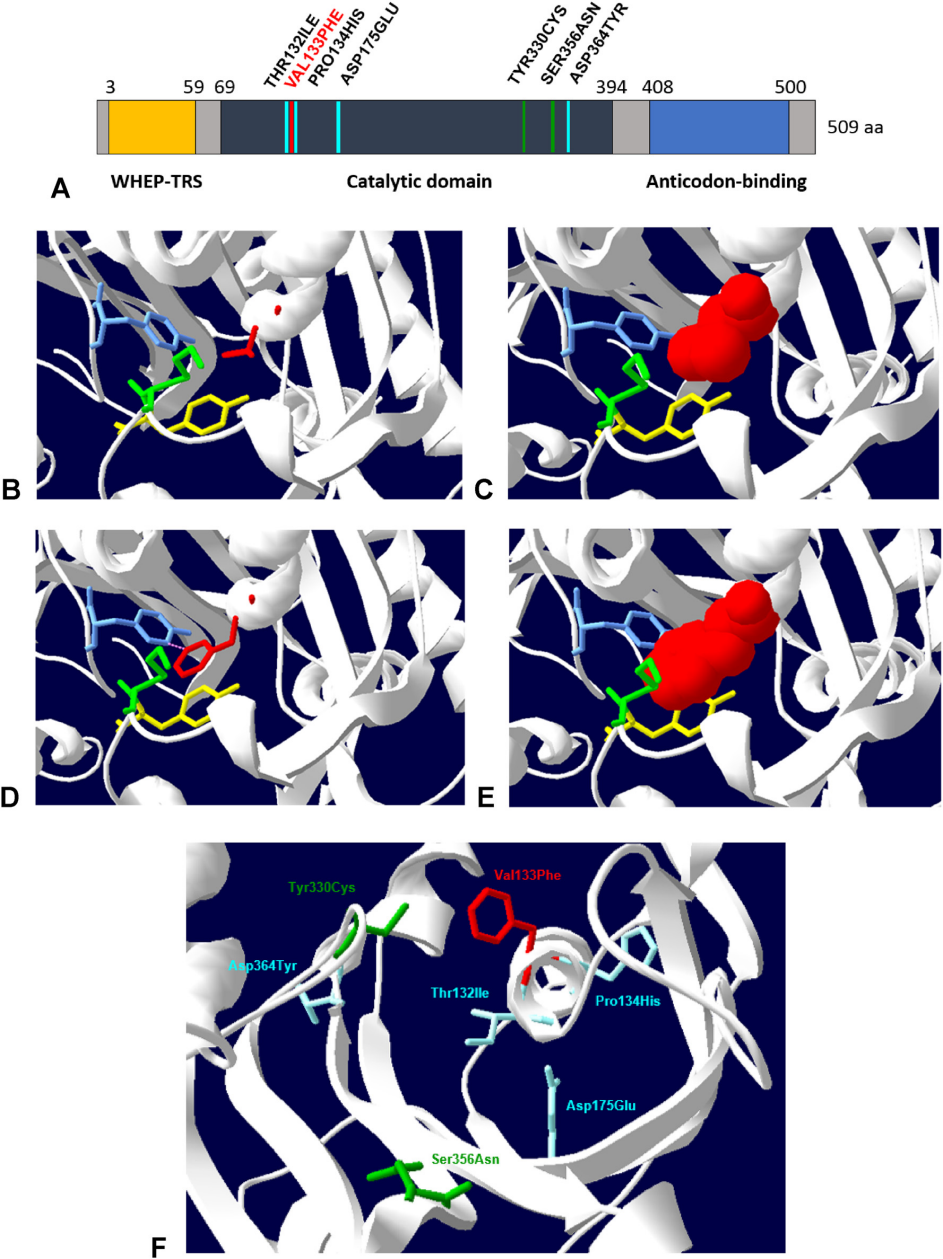
Dominios de la proteína histidil-ARNt sintetasa (HARS). (A) Dominios de HARS. Se incluyen las variantes descritas en la literatura resaltando en rojo la sustitución V133F descrita en este artículo. (B–E) Localización de estas variantes asociadas a CMT en el sitio activo de HARS (PDB 4PHC). Las diferentes sustituciones aparecen en estructura 3D sobre la representación de la estructura secundaria de la proteína. En (B–E) se muestra el impedimento estérico que aparece en el residuo 133 del centro catalítico cuando la proteína presenta la sustitución V133F (Phe133, imágenes D–E) frente a cuando aparece en su forma nativa (Val133, imágenes B–C). En (B) y (D) se muestran las representaciones 3D de la variante V133F (rojo) y aminoácidos del centro catalítico con los que interacciona (amarillo-Tyr107, verde-Lys106 y azul-Tyr330). En (C) y (E) se ha incluido la representación de los radios de Van der Waals en superficies 3D. En (F) se recogen el resto de mutaciones descritas en la literatura (en azul las descritas en Brozkova et al. y en verde las de Abbott et al.) [12, 13]. Las imágenes (B–F) se han realizado con el programa SPDBviewer.

Además, el análisis bioinformático predice que altera la estructura o función de la proteína codificada por el gen *HARS* y programas de modelado tridimensional sugieren que la sustitución Val133Phe ocasiona impedimentos estéricos con los residuos cercanos Lys106, Tyr107 y Tyr330 (Figura 3). El programa Human Splicing Finder predice que origina un splicing aberrante, dando lugar a alteraciones en el tránscrito de mRNA.

En aquellos casos en los que el cuadro clínico no puede explicarse con el análisis de las mutaciones más frecuentemente asociadas a la patología estudiada, el uso de NGS, exoma clínico, permite descubrir variantes raras o previamente no descritas responsables del fenotipo del paciente, solas o en combinación con otras, así como hallazgos incidentales. En nuestro caso, el exoma clínico ha permitido identificar la mutación Gly396Asp en el gen *MUTHY* en heterocigosis que revela la predisposición del paciente a padecer cáncer de colon. A su vez, este descubrimiento ha permitido ampliar el estudio genético a sus progenitores llegando a diagnósticos tempranos de CMT2W en la madre lo que se justificaría por una penetrancia incompleta y la diferente expresividad clínica entre miembros de una misma familia y polipomatosis adenomatosa familiar en el padre, permitiendo realizar un adecuado asesoramiento genético y establecer las medidas preventivas correspondientes.

Puntos de aprendizaje

- La presencia de variantes en el gen *HARS* se asocia, con un patrón de herencia autosómico dominante, a la enfermedad de CMT axonal tipo 2W.
- La enfermedad de CMT axonal tipo 2W es un trastorno neurológico caracterizado por una neuropatía periférica, que afecta principalmente a las EEII y provoca dificultades en la marcha y deterioro sensorial distal.
- El empleo de técnicas de secuenciación masiva, NGS, ha permitido un aumento exponencial en el reconocimiento de las mutaciones implicadas en las neuropatías hereditarias como la enfermedad de CMT.
- El exoma clínico es una herramienta que permite ir del genotipo al fenotipo en aquellos cuadros clínicos de etiología no filiada.
- La implementación de las técnicas de secuenciación masiva, exomas clínicos, en ocasiones implica la presencia de hallazgos incidentales con implicaciones en el asesoramiento genético del paciente y sus familiares.

## Supplementary Material

Supplementary Material Details

## Material Suplementario

La versión en línea de este artículo ofrece material suplementario (https://doi.org/10.1515/almed-2020-0020).
