# Supplementary material for: Variante en gen HARS detectada en exoma clínico: etiología de neuropatía periférica tras más de 20 años sin diagnóstico
Source: Adv Lab Med. 2020 May 19;1(4):20200020. [Article in Spanish] doi: 10.1515/almed-2020-0020 (PMC10197309; doi:10.1515/almed-2020-0020)
Supplement: Supplementary file 1 — Supplementary Material Details [file j_almed-2020-0020_suppl.docx]

**Anexo 1. Panel NGS de 34 genes asociados a la enfermedad de Charcot-Marie-Tooth (Sistemas Genómicos, ASCIRES, Valencia, España).**

Genes estudiados: *AARS, ARHGEF10, DNM2, DYNC1H1, EGR2, FGD4, FIG4, GARS, GDAP1, GDAP1L1, GJB1, HK1, HSPB1, HSPB8, KARS, KIF1B, LITAF, LMNA, LRSAM1, MED25, MFN2, MPZ, MTMR2, NDRG1, NEFL, PRX, PMP22, PRPS1, RAB7A, SBF2, SH3TC2, SPTLC1, TRPV4, YARS*.
